# MSafe: Falls Risk App for Individuals With Multiple Sclerosis

**DOI:** 10.1093/geroni/igaf122.244

**Published:** 2025-12-31

**Authors:** Katherine Hsieh, Deborah Backus, Jon Sanford

**Affiliations:** Georgia State University, Atlanta, Georgia, United States; Shepherd Center, Atlanta, Georgia, United States; Georgia State University, Atlanta, Georgia, United States

## Abstract

Multiple Sclerosis (MS) is a chronic, autoimmune, disease of the central nervous system with the highest prevalence in adults aged 55 years and older. MS is a dynamic condition with symptoms fluctuating daily to weekly, placing people with MS at a high risk of falls. Symptom variability makes it challenging for older adults with MS to manage their fall risk. Our research team developed MSafe, a mobile health application designed to help older adults with MS manage their fall risk. In this co-design process, we interviewed 6 MS experts to identify important factors and components to include in the app. Experts identified key risk factors to incorporate. We then collaborated with an app development team to design MSafe, which evaluates multifactorial fall risk through assessment of: 1) patient reported outcomes (e.g., demographics, sensory function, fatigue), 2) environmental hazards, 3) physical function, and 4) cognition. MSafe displays individualized results compared to clinical thresholds. To assess usability, 10 older adults with MS independently completed MSafe, followed by semi-structured interviews that were video recorded, transcribed, and analyzed thematically. Three themes emerged: 1) ease of use, 2) fall risk feedback, and 3) bilateral adaptations. Participants reported that MSafe was intuitive and easy to follow. Following the assessment, participants expressed interest in conducting repeated assessments to track changes over time. Last, those using bilateral assisted devices requested a phone holder for dynamic tasks. These findings suggest potential for independent, fall risk assessments with mobile technology and inform next iteration of MSafe prior to home evaluation.

